# Castor Oil and Epsom Salts Made Palatable

**Published:** 1904-12

**Authors:** 


### CASTOR OIL AND EPSOM SALTS MADE PALATABLE.

Valuable therapeutic remedies are castor oil and Epsom salts but their taste is very much against them. Dr. E. P. Carlton, however, has been administering these drugs for some time without friction or bribery, and giving them continuously to delicate and sensitive patients, without the latter suspecting the fact. The formulas of his preparations are as follows:

OLEUM RICINI DULCE.

| | |
|---|---|
| Vanillin | gr. xx |
| Olei. Menth. Pip | ʒ j |
| Garantose (Saccharin) | ʒ jss |
| Alcohol | ℥ jii |
| Tinct. Persionis | ℥ ss |
| Olei. Ricini, q.s. ad. one-half gallon. | |

*Directions for Mixing.*—Dissolve the vanillin, oil of peppermint, and garantose in the alcohol. Add the tincture of cudbear to the oil and shake thoroughly. Finally unite the two mixtures. [Instead of the plain garantose, it is better to use the water-soluble garantose "crest."]

This mixture keeps well, looks nice, is pleasant to take, does not leave a bad after-taste; requires no urging to get it down and therefore, for all practical purposes, is a *disguised oil.*

LIQUOR MAGNESII SULPHATIS COMP.

| | |
|---|---|
| Magnesii Sulphatis | ℥ xxxii |
| Tinct. Cardamomi Comp | ℥ ii |
| Vanillin | gr. xx |
| Garantose "Crest" Merck | ℥ ii-iv |
| Alcohol | ℥ i |
| Glycerin | ℥ ii |
| Coffee, roasted and ground | ℥ ii |
| Aquæ, q.s. ad. one-half gallon. | |

*Directions for Mixing.*—Stir the ground coffee in one-half gallon of boiling hot water, and allow it to stand for ten to twenty minutes. While this is still hot add enough of it to the magnesium sulphate to make about three and one-half pints. Dissolve the vanillin in the alcohol, add the glycerin to it and then the cardamom. When the first solution has cooled somewhat add the second mixture to it. After shaking thoroughly add the garantose ("crest") and enough of the coffee infusion to make one-half gallon. Finally filter through a covered filter.

An ounce of this solution contains one-half an ounce of magnesium sulphate.

This solution keeps well, has a dark, whisky-like color, a nutty odor, and is easy to take, warm or cold. The author dilutes it with twice its volume of water at the time of administration.—*Medical News, October 8, 1904.*

